# Blastomycose pulmonaire

**DOI:** 10.11604/pamj.2020.36.220.21829

**Published:** 2020-07-28

**Authors:** Fatma Chermiti Ben Abdallah, Imène Bachouch, Nidhal Belloumi, Marwa Kacem, Mouna Mlika, Faouzi El Mezni, Soraya Fenniche

**Affiliations:** 1 Service de Pneumologie IV, Faculté de Médecine de Tunis, Hôpital Abderrahmen Mami Ariana, Tunis, Tunisie,; 2 Service d’Anatomie Pathologique, Faculté de Médecine de Tunis, Hôpital Abderrahmen Mami Ariana, Tunis, Tunisie

**Keywords:** Blastomycose, biopsie, *Blastomyces dermatitidis*, antifongique, Blastomycosis, biopsy, Blastomyces dermatitidis, antifungal

## Abstract

La blastomycose est une maladie fongique rare en Afrique, due souvent à l’inhalation de « Blastomyces dermatitidis ». La forme pulmonaire est la manifestation clinique la plus fréquente, pouvant aller de la forme asymptomatique jusqu’à la forme rapidement mortelle. Nous rapportons l’observation d’un patient tunisien âgé de 35 ans sans antécédents médicaux, hospitalisé pour une toux chronique, des douleurs basithoraciques bilatérales, fièvre et un amaigrissement. L’examen clinique a objectivé la fièvre ainsi qu’une tuméfaction sous cutanée para-vertébrale gauche en regard de la dixième vertèbre thoracique (T10). L’imagerie thoracique a objectivé des opacités alvéolaires et nodulaires bilatérales excavées par endroit. La recherche de bacille de Koch (BK) dans les expectorations était négative à l’examen direct et à la culture. La fibroscopie bronchique était normale. L’étude anatomopathologique de la biopsie de la masse dorsale a conclu à une blastomycose et le diagnostic a été confirmé par le résultat des cultures des fragments biopsiques de la masse sus décrite. Un traitement antifongique à base d’itraconazole a été instauré avec une amélioration clinique et radiologique. Ce cas illustre la difficulté diagnostique que peut poser la blastomycose, notamment, avec la tuberculose dans notre pays, d’où le retard thérapeutique.

## Abstract (en)

Blastomycosis is a rare fungal disease in Africa which is often due to inhalation of “Blastomyces dermatitidis”. Pulmonary blastomycosis is the most common clinical manifestation which presents with a variety of clinical features, ranging from asymptomatic to rapidly fatal. We report the case of a Tunisian patient aged 35 years with no previous medical history, hospitalized with chronic cough, bilateral basithoracic pain, fever and weight loss. Clincal examination showed fever and left paravertebral subcutaneous swelling next to the tenth thoracic vertebra (T10). Chest imaging objectified bilateral alveolar and nodular opacities with excavations in some places. Sputum stain for Koch bacillus (BK) was negative (direct examination and culture). Bronchial fibroscopy was normal. Anatomopathological examination of dorsal mass biopsy revealed blastomycosis. The diagnosis was confirmed by cultures of the biopsic fragments of the mass. Antifungal therapy with itraconazole was started with clinical and radiological improvement. This case study highlights challenges in the diagnosis of blastomycosis in our country, in particular when lesions mimick tuberculosis; hence delayed therapy.

## Introduction

La blastomycose est une infection fongique endémique dans certaines régions de l’Amérique du nord (les vallées de l'Ohio-Mississippi, le nord du Midwest, le nord de l'État de New York, et le sud du Canada). Son incidence est très variable allant de 1,4 à 40 cas pour 100 000 habitants selon les régions [1]. Cette infection est plus rare au Moyen-Orient et en Afrique et survient souvent sous formes de cas sporadiques, même chez les personnes immunodéprimées [2]. La blastomycose est due à l’inhalation d’un champignon dimorphique « *Blastomyces dermatitidis* », dans la majorité des cas. Dans les poumons, les spores inhalées se transforment en levures invasives dont la taille est de 15 à 20 μm et forment des bourgeons caractéristiques à base large. L’infection peut rester au niveau des poumons, donnant un tableau de pneumonie, ou disséminer par voie hématogène et être à l’origine de localisations extrapulmonaires: tissus sous-cutanés, cerveau, os [1, 3].

La présentation clinique dépend de la localisation. Dans tous les cas, les signes généraux sont fréquents. Dans la forme pulmonaire, il s’agit d’un tableau de pneumonie, souvent d’évolution insidieuse. Les données de l’imagerie thoracique ne sont pas spécifiques. La confirmation de la blastomycose est essentiellement bactériologique, par la mise en culture des différents types de prélèvements. Le traitement dépend de la gravité de la maladie. L’itraconazole est réservé aux formes légères à modérées et l’amphotéricine B est indiquée dans les infections graves avec mise en jeu du pronostic vital [1, 4]. Nous présentons un cas de blastomycose, qui illustre la difficulté diagnostique de cette pathologie dans notre pays, vu sa rareté et son tableau clinique non spécifique, pouvant simuler une tuberculose pulmonaire, d’où le retard diagnostique et thérapeutique.

## Patient et observation

**Figure 1:**
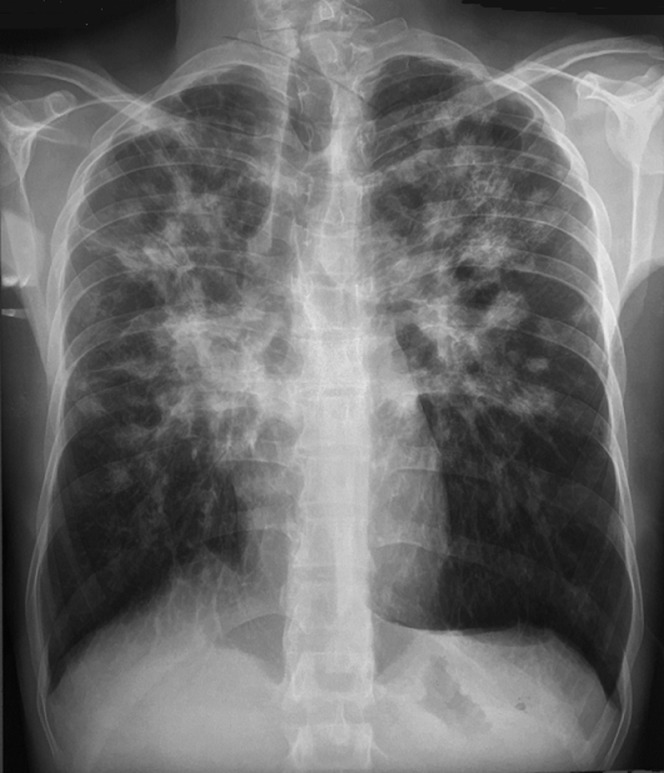
radiographie thoracique de face montrant des opacités alvéolaires et nodulaires confluentes prédominant au niveau des régions supérieures des deux champs pulmonaires

Patient R.A, âgé de 35 ans, tabagique actif à 25 PA, originaire du nord-ouest de la Tunisie, vivant dans un milieu urbain, n’ayant jamais voyagé et ayant travaillé comme préparateur dans un laboratoire de lycée secondaire avec notion de manipulation d’organes vitaux d’animaux. Il a été hospitalisé dans notre service pour une toux sèche chronique, des douleurs thoraciques bilatérales, un amaigrissement non chiffré et une fièvre. L’examen a montré un patient fébrile à 38°C et l’un examen pleuro-pulmonaire était sans anomalies. A l’examen des aires ganglionnaires, il n’y avait pas d’adénopathies périphériques. La flèche hépatique était à 10cm et il n’y avait pas de splénomégalie. Par ailleurs, nous avons noté la présence d’une masse para-vertébrale gauche de 7cm en regard de T10 ferme, indolore, sans signes inflammatoires cutanés en regard. La radiographie du thorax de face, a montré des opacités alvéolaires et nodulaires confluentes prédominant au niveau des régions perihilaires et supérieures des deux champs pulmonaires (Figure 1). La biologie a montré une légère hyperleucocytose à 10500/mm^3^ (PNN: 7890/mm^3^, PNE: 280/mm^3^) et un syndrome inflammatoire biologique (VS accélérée à 107mm à la première heure et CRP à 33mg/l). Le bilan rénal et hépatique était normal. Le diagnostic de tuberculose pulmonaire a été évoqué en premier mais la recherche de bacille de Koh (BK) dans les crachats et dans le liquide bronchique était négative à l’examen direct et à la culture.

**Figure 2:**
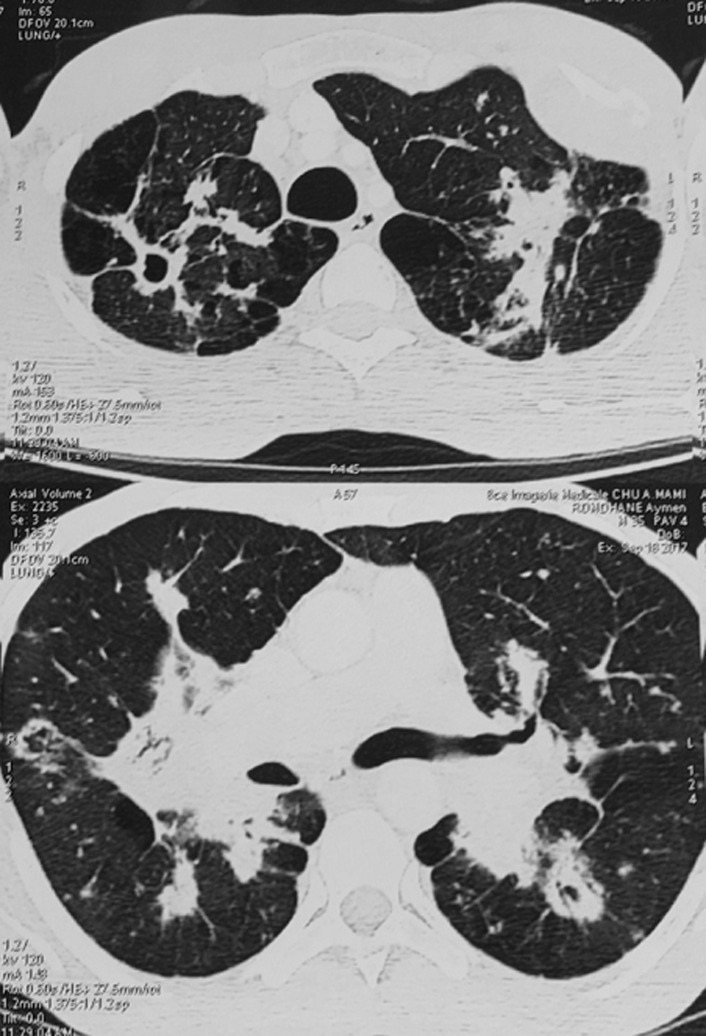
TDM thoracique en fenêtres parenchymateuses objectivant des condensations et nodules de taille variables, excavés par endroit, et des lésions d’emphysème centrolobulaire prédominant aux apex pulmonaires

**Figure 3:**
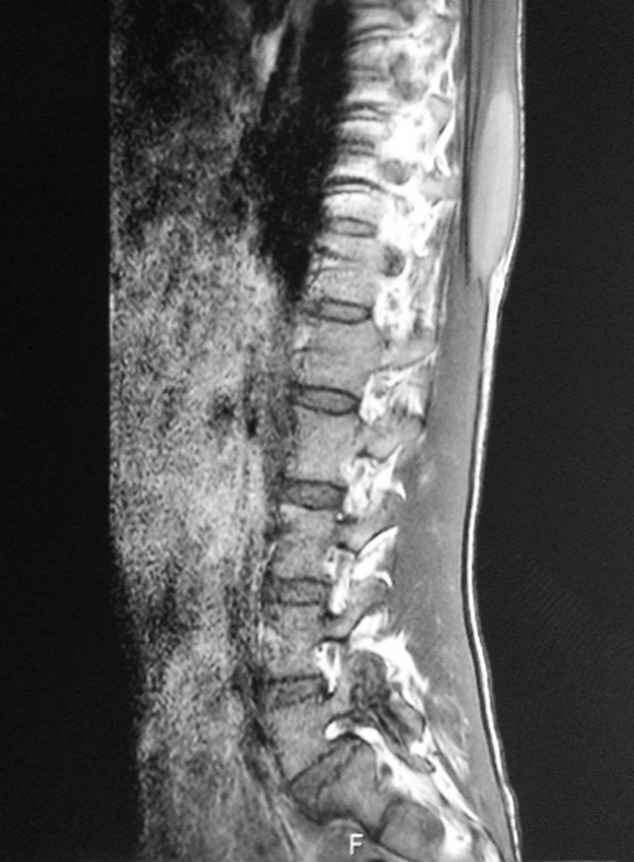
IRM montrant une masse des muscles para-vertébraux (indiquée par la flèche rouge) avec interruption du ligament inter-épineux

**Figure 4:**
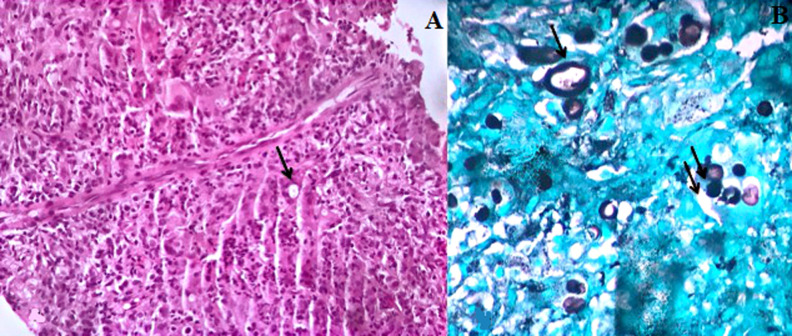
A) examen microscopique montrant des lésions granulomateuses avec la présence au niveau des cellules géantes, de levures (flèche) (HEx250); B) coloration argentique au Gomori-Grocott montrant des levures à paroi épaisse (flèche) avec des bourgeonnements unipolaires à base large (HEx400)

La tomodensitométrie (TDM) thoracique a objectivé des condensations et des nodules de taille variables et confluents, notamment en péri-hilaires, excavés par endroit, un collapsus non aéré du lobe moyen et des lésions d’emphysème centrolobulaire prédominant aux apex pulmonaires. (Figure 2). L’échographie des parties molles en regard de T10 a trouvé une collection des muscles para-vertébraux. Afin de mieux caractériser cette lésion, nous avons complété par une imagerie par résonance magnétique (IRM) rachidienne qui a confirmé la présence d’une masse des muscles para-vertébraux avec interruption du ligament inter-épineux (Figure 3). La fibroscopie bronchique était normale et les biopsies bronchiques étagées étaient d’aspect inflammatoire non spécifique. Une biopsie de la masse sous cutanée, a été réalisée sous contrôle scannographique et l’étude anatomopathologique a montré un tissu fibreux et adipeux siège d’assez nombreux granulomes épithélioïdes et giganto-cellulaires sans nécrose associée. On notait au sein des cellules géantes, la présence de levures de grande taille, difficiles à analyser à l’hématoxyline éosine. L’imprégnation argentique de Gomori-Grocott a permis de souligner l’aspect en double contour de la paroi des levures ainsi qu’un bourgeonnement unipolaire s’effectuant sur une base large. L’aspect cadrait avec une blastomycose (Figure 4). L’examen microbiologique des crachats et du liquide bronchique n’a pas objectivé de blastomyces. Néanmoins, la culture des fragments biopsiques de la masse paravertébrale était positive avec présence de colonies blanches et cotonneuses. Nous avons retenu le diagnostic de blastomycose pulmonaire avec localisation musculaire.

Un traitement antifongique à base d’amphotéricine B par voie intraveineuse lente à la dose initiale de 0,1mg/kg/jour. L’évolution a été marquée six jours après le début d’administration du traitement, par l’aggravation de la fièvre à 39°C, une tachycardie à 150 battements par minute, une baisse de la tension artérielle et une polypnée à 22 cycles par minute et une désaturation artérielle à 90%. Une réaction allergique sévère, stade 3, à l’amphotéricine B a été suspectée, conduisant à l’arrêt immédiat de l’amphotéricine B, une oxygénothérapie par sonde nasale, un remplissage vasculaire par un litre de sérum salé isotonique, avec injection intraveineuse de 200mg d’hémisuccinate d’hydocortisone et la prescription d’un antihistaminique avec une bonne évolution clinique. Le patient a été par la suite mis sous itraconazole, à la dose de 200mg trois fois par jour pendant deux semaines puis 200mg deux fois par jour pendant 6 mois. A deux mois de traitement, nous avons noté une nette amélioration clinique avec une disparition de la fièvre et des signes fonctionnels respiratoires et une diminution en taille de la masse para-vertébrale passant de 7cm à 4cm de diamètre. La radiographie thoracique a montré un début de nettoyage avec apparition de signes de rétraction pulmonaire.

## Discussion

La blastomycose, connue aussi sous le nom de maladie de Gilchrist est une infection fongique relativement rare, endémique en Amérique du nord et au Canada avec des cas sporadiques décrits en Angleterre, Pologne, Moyen-Orient, en Inde et en Afrique [1]. Au Maghreb, elle est rare. Environ une quinzaine de cas ont été décrits au Maroc, en Algérie et en Tunisie [5]. Son incidence est variable selon les pays, mais, dans les zones très endémiques pour cette maladie, un taux d’incidence de 40 cas /100 000 habitants, a été rapporté [1]. La maladie est due à un champignon dimorphique: le *Blastomyces dermatitidis* (BD). Il existe sous forme mycélienne dans l’environnement ou à 25°C et sous forme de levure dans les tissus humains ou à 37°C [1]. Il s’introduit chez l’homme essentiellement par inhalation. L’inoculation transcutanée est rare, survenant accidentellement après une blessure chez des sujets à professions exposées (vétérinaires, anatomopathologistes) ou après manipulation de produits de laboratoire ou d’autopsie plus rarement après morsure, ou égratignure par un animal contaminé. Chez notre patient, les deux modes de transmission ont été évoqués vu la notion de manipulation d’organes d’animaux. Mais, l’existence de deux localisations rend la voie inhalée la plus probable chez notre patient. La transmission interhumaine n’a pas été prouvée [2]. Après inhalation, les spores initient une réponse inflammatoire, au niveau du poumon, qui aboutit à la formation de granulomes au bout de 3 semaines à 3 mois. Chez certains malades, une dissémination hématogène peut survenir, donnant lieu à des localisations extrapulmonaires. Parmi ces formes, les atteintes cutanées, sous-cutanées, osseuses, et génito-urinaires sont les plus fréquentes [6]. L’atteinte musculo-squelettique, tel le cas de notre malade, est extrêmement rare [7].

Les manifestations cliniques de la blastomycose sont polymorphes au point de l’avoir qualifiée du « meilleur prétendant: the great pretender » [8]. La forme clinique la plus fréquente étant la forme pulmonaire [1]. Le patient présente souvent un tableau insidieux dominé par les signes généraux: une fièvre, des sueurs nocturnes et un amaigrissement. Les signes respiratoires se résument souvent en une toux sèche ou productive, comme cela a été décrit chez notre patient. La dyspnée et la douleur thoracique sont rares. Un début aigu voire même foudroyant, réalisant un syndrome de détresse respiratoire aigüe (SDRA), d’évolution mortelle a été décrit [8]. Les signes extra-respiratoires dépendent de la localisation de la maladie. Contrairement à l’histoplasmose et la cryptococcose, la blastomycose atteint plutôt des patients immunocompétents. Les cas décrits chez les sujets immunodéprimés notamment sidéens sont des formes sévères (SDRA, miliaire) avec une mortalité dépassant 50% [1]. Notre patient est immunocompétent et sa sérologie VIH était négative.

A l’imagerie thoracique, la blastomycose pulmonaire peut se présenter sous forme d’infiltrat ou d’opacités alvéolaires et interstitielles bilatérales locales ou diffuses. Un aspect de bronchopneumonie bilatérale ou d’opacité suspecte de malignité ont été décrits [4]. Les nodules, lorsqu’ils existent, sont de taille variable et peuvent être excavés. Des adénopathies médiatisnales, une atteinte pleurale ou une condensation lobaire ou segmentaire peuvent se voir également [1, 3, 4, 7]. Les formes excavées font évoquer plutôt une tuberculose pulmonaire, surtout dans les pays endémiques pour cette maladie, d’où le retard diagnostique. Notre malade avait une atteinte bilatérale avec des nodules excavés par endroit, nous avons ainsi pensé à une tuberculose pulmonaire en premier mais les cultures de BK dans les expectorations et le liquide bronchique étaient négatives. Une origine néoplasique primitive pulmonaire ou secondaire a été aussi évoquée, mais, il n’y avait pas de signes cliniques en faveur d’un néoplasie et les biopsies bronchiques étagées étaient négatives. Le manque de spécificité des manifestations cliniques et radiologiques, rend souvent le diagnostic de blastomycose tardif entrainant ainsi un retard thérapeutique, surtout dans les pays ou cette pathologie est rare comme le nôtre. Ce diagnostic doit être suspecté devant une symptomatologie respiratoire persistante dans un contexte fébrile, malgré une antibiothérapie probabiliste bien conduite et en l’absence d’autres diagnostics, particulièrement la tuberculose. Le délai de confirmation diagnostique chez notre malade était de cinq mois. Ce délai est expliqué par la forte suspicion initiale de la tuberculose pulmonaire entrainant la multiplication des examens bactériologiques dans les expectorations et le liquide bronchique, qui étaient tous négatifs à l’examen direct et à la culture.

La confirmation diagnostique de la blastomycose est bactériologique par la mise en culture des prélèvements de tissus infectés, mettant en évidence, un aspect caractéristique [1]. L’examen histopathologique des tissus infectés par le BD, est également d’un grand apport diagnostique. L’examen standard à l’hématoxyline éosine ne permet pas d’apprécier les détails des levures. Cependant, la coloration argentique de Gomori-Grocott permet de souligner certains critères distinctifs comme la double paroi et le bourgeonnement unipolaire à base large [1, 5]. Ces critères étaient observés dans notre observation et ont permis de distinguer les levures de blastomyces comme la cryptococcose. Cette dernière se distingue par une taille variable, l’absence de double capsule et un bourgeonnement à base étroite. Chez notre malade la biopsie transpariétale sous scanner de la masse dorsale a permis d’évoquer le diagnostic en montrant des levures à paroi épaisse, réfringente avec une base large d’implantation du bourgeonnement caractéristique. Le résultat des cultures a permis la confirmation diagnostique, qui reste jusqu’à ce jour le gold standard. La sérologie bien que spécifique, n’est pas très sensible. Lorsqu’elle elle est positive, elle constitue un argument de plus pour le diagnostic [9].

Les indications thérapeutiques dépendent, surtout, de la sévérité de l’atteinte et du statut immunitaire du patient [2]. Le traitement des formes sévères, pouvant mettre en jeu le pronostic vital, repose initialement, sur l’amphotéricine B en intraveineux [2]. La dose varie de 3 à 5mg/kg une fois par jour pour la forme liposomale et de 0,7 à 1mg/kg une fois par jour pour l’amphotéricine B désoxycholate, pendant deux semaines en moyenne. Le relais est assuré par l’itraconazole par voie orale, à la dose de 200mg trois fois par jour les trois premiers jours puis deux fois par jour pendant 6 à 12 mois. Pour les formes légères ou modérées, le traitement repose sur l’itraconazole aux mêmes doses sus décrites. Le rôle des autres antifongiques (voriconazole, posaconazole, isavuconazole) n’est pas encore défini [9]. Chez notre patient, nous avons opté pour un traitement initial par amphotéricine B vu les signes cliniques très marqués, la bilatéralité de l’atteinte pulmonaire et l’existence d’une autre localisation, témoignant d’une dissémination très probablement hématogène. Ce traitement a été rapidement interrompu suite à la réaction allergique sévère. Nous avons prescrit par la suite, l’itraconazole à la dose de 200mg deux fois par jour et l’évolution était lentement favorable. Le pronostic dépend du terrain, de la gravité et du siège de l’infection. En effet, dans les formes sévères, réalisant un tableau de SDRA, la mortalité varie de 50 à 89% chez les patients immunodéprimés [1]. Chez notre patient, l’amélioration clinique a été favorable mais le nettoyage radiologue n’était que partiel avec apparition de signes de rétraction radiologique. Des travaux concernant la possibilité de vaccination contre la blastomycose ont été rapportés, la majorité ont porté sur la protéine de surface BAD-1 du BD avec de bons résultats sur des modèles animaux [10]. Il n’existe pas encore des mesures prévention spécifique, mais des mesures de protection doivent être entreprises en cas d’activité pratiquée en contact avec le sol humide ou en zone d’endémie [10].

## Conclusion

Le diagnostic de la blastomycose pulmonaire, pathologie rare en Tunisie, peut être déroutant simulant d’autres pathologies, essentiellement, la tuberculose. Afin de réduire le délai diagnostique, la blastomycose pulmonaire, doit être suspectée devant toute pneumonie d’évolution insidieuse avec négativité de l’étiologique. La confirmation est, avant tout, microbiologique par la mise en culture des tissus infectés. L’amphotéricine B et l’itraconazole représentent les principaux antifongiques efficaces dans cette pathologie.
